# Vitamin Supplementation in Pre-Pregnancy and Pregnancy among Women—Effects and Influencing Factors in Romania

**DOI:** 10.3390/ijerph19148503

**Published:** 2022-07-12

**Authors:** Calin Avram, Oana Maria Bucur, Ancuța Zazgyva, Laura Avram, Florina Ruta

**Affiliations:** 1 George Emil Palade University of Medicine, Pharmacy, Science and Technology, 38 Gh. Marinescu St., 540139 Targu Mureș, Romania; ancuta.zazgyva@umfst.ro (A.Z.); florina.ruta@umfst.ro (F.R.); 2 Dimitrie Cantemir University, 3-5 Bodoni Sandor St., 540545 Targu Mureș, Romania; eleonora_avram@yahoo.com

**Keywords:** vitamins, pregnancy, dietary supplements, post-partum women, folic acid, Romania

## Abstract

Introduction: The aim of the study was to identify the consumption of vitamin and folic acid supplements before and during pregnancy in a group of post-partum women (Romanian, Hungarian, and Roma) from Mureș County, Romania, and the influence of socio-economic and behavioral factors on the consumption of vitamins. Materials and Methods: This cross-sectional questionnaire-based study included 1278 post-partum women (during the three days of hospitalization for birth), average age 29.5, registered for giving birth in the three hospitals in Mureș County, 2015–2016. Results: In our sample, 69.58% of the interviewed women did not use any vitamin and folic acid supplements before pregnancy, while 30.70% did not use vitamin supplements during pregnancy. The lack of vitamin supplementation during pregnancy was associated with the low birth weight (<2500 g) of newborns (OR = 2.4, 95% CI [1.6–3.8]) and birth at under 36 weeks of gestation (OR = 0.5, 95% CI [0.2–0.8]). Conclusion: The use of vitamin supplements, including folic acid, continues to be deficient among Romanian women before getting pregnant, as well as during their pregnancy. We observed a lack of vitamin supplementation for pregnant women even if they were influenced by risk factors. This highlights the importance of promoting the benefits of vitamin supplementation equally among all subjects.

## 1. Introduction

According to previous reports, more than 300,000 deaths occurred in the first days of life and one of the most important causes was the neural tube defect (NTD) [1]. This is attributed to the need for nutrients during pregnancy but is most important in the first trimester of pregnancy [2]. Of course, both before and after conception, the nutritional status is very important; it affects both the development of the fetus and its growth. An important element is folic acid, and its deficiency is very common among women who want to get pregnant but may also lack other micronutrients such as Fe, Iodine, and vitamin D [1]. 

Vitamin deficiencies during or before pregnancy can affect the health of both mother and fetus [3,4]. For example, if a woman experiences prolonged vomiting during pregnancy, she is at risk of developing a thiamine deficiency [5]. Recent research proposes that deficiencies of some nutrients are associated with problems in fetal growth and development, complications during pregnancy, and alterations in the later development of children [6], but also changes that influence the maternal intestinal microbiome and, potentially, the fetal microbiome [7,8]. Similarly, low levels of vitamin B6 before pregnancy have been associated with an increased risk of preterm birth and miscarriage, in a study on a group of women from Vancouver (Canada) [9]. Insufficient vitamin B12 and folate consumption may increase the risk of premature birth (<37 week) and low birth weight (<2500 g) [10], while later on (during childhood) it may adversely affect cognitive and motor development [10]. 

Biologically, active forms of vitamin B9 (folic acid and folates) are essential for cell division, erythropoiesis, and fetal growth and development. Folate or vitamin B9 is an essential vitamin but cannot be synthesized by organism, and so we must take it either from foods that contain this element naturally or through dietary supplements prescribed by a doctor [11]. Since September 2013, the Center for Disease Control and Prevention (CDC) in the USA has recommended that women of childbearing age take a supplement of 400 micrograms of folic acid a day, starting at least four weeks before conception, as well as during the first trimester of pregnancy, in order to reduce the risk of congenital anomalies [12]. Folate deficiency in pregnant women was associated with increased rates of congenital anomalies, including neural tube affection at birth, cardiovascular disease, macrocytic anemia, and carcinogenic processes [13,14].

Studies have shown that folic acid intake during pregnancy reduces NTD, but this is only effective in women who are planning a pregnancy or who are in the first period of pregnancy. In the USA and Canada, national programs have been implemented to recommend that pregnant women consume folic acid. This intake should be administered daily at least 2–3 months before conception and throughout pregnancy and even in the postpartum period [11,15]. 

A study conducted in the EU estimates that between 1998 and 2017 there was a prevalence of 0.92 births that presented NTD over the years. There are few studies on vitamin consumption among pregnant women in Romania. Pregnant women identify the need for vitamins only at prenatal visits. In the case of pregnant women, a healthy diet [16,17] does not exclude the possibility of imbalances of some essential nutrients. As such, deficiencies can be covered with prenatal vitamins. In particular, demands for folate increase during pregnancy, which is also required for the growth and the development of the fetus. Folate deficiency has been associated with abnormalities in both mothers (anemia, peripheral neuropathy) and fetuses (congenital abnormalities) [18]. A study conducted in a region of Romania in 2010 on a sample of 400 pregnant women identified that 48.0% of participants used folic acid, 45.3% used iron supplements, and 68.0% used multivitamins during pregnancy [19].

The aim of the study was to identify the consumption of vitamin supplements (including folic acid) before and during pregnancy in a group of post-partum women from Mureș County, and the influence of socio-economic (rural residence, marital status, level of education) and behavioral factors (coffee consumption, alcohol consumption, smoking) on the consumption of vitamins, along with the impact on the evolution of the pregnancy and on the newborn’s parameters. We hope that a study like this can help identify pregnant women who have low folic acid intake, which in turn will help develop appropriate programs to improve vitamin intake in pregnant women or those who want to become pregnant. These programs will be very important for pregnant women who have been identified with low folic acid intake.

## 2. Materials and Methods

### 2.1. Data Collection

This cross-sectional questionnaire-based study included 1278 post-partum women who gave birth in three hospitals. The hospitals were located in Tîrgu Mures, a city in the Transylvanian region in Romania with a population of 130,000 inhabitants. About 2500 children are born annually, in each of these hospitals. Data were collected from March 2015 to March 2016, using convenience sampling. During the period of our study, all the women hospitalized for childbirth were included in our study (obviously only those who agreed to this study), they were interviewed by a person trained for this purpose, and the questionnaire was completed by the women in the three days of post-partum hospitalization. The interviews were made by trained staff, based on a validated questionnaire consisting of 109 items (of which 50 directly related to smoking attitudes) and a convenient method of data collection. The study was conducted to identify pregnant women who smoke; however, there were questions that followed other important elements in the life of a pregnant woman. 

The inclusion criteria in this study were that pregnant women were hospitalized for childbirth; the exclusion criteria were for pregnant women who reported an irregular intake of vitamins and folic acid.

In Romania, almost all women are hospitalized for at least 3 days post-partum, during which time the newborn is immunized against tuberculosis and hepatitis B. However, it is very uncommon to leave the hospital earlier [19].

The research protocol was approved by the ethics committee at the ‘’George Emil Palade’’ University of Medicine, Pharmacy, Science and Technology of Târgu Mureș and the study was conducted in accordance with the Helsinki Declaration.

### 2.2. Measurements

Although the study was conducted to identify pregnant women who smoke, various associations could be made to identify the phenomena that are found in the lives of pregnant women, such as the consumption of vitamins during pregnancy. The questionnaire did not require an answer regarding the period of time through which vitamins were taken before conception. The questionnaire was validated (or performed all the steps necessary to validate the questionnaire) before distribution to pregnant women.

The following parameters were evaluated: vitamin supplements and folic acid supplements consumption before and during pregnancy. The following questions were used: “Did you use vitamin supplements (vitamins and folic acid) before conception?” and “Have you regularly taken vitamin supplements during pregnancy?”. 

The methodology excluded respondents who declared vitamin supplementation but had an irregular administration. Variables collected were: age, residency, marital status, level of education, family income, number of prenatal visits to the physician, weight before pregnancy, and weight gain during pregnancy (the values were self-reported for pre-pregnancy weight, while weight at birth was measured at hospital admission). Regarding the newborns, the following parameters were followed: birth weight and length, head circumference at birth, and gestational age at birth. Women’s weight was self-reported for pre-pregnancy weight, and for birth weight it was measured at hospitalization during pregnancy. Based on the collected data, we tried to identify whether vitamin intake has an effect of weight gain in pregnant women. All the information related to the BMI (Body Mass Index) of women before pregnancy, the weight gain during pregnancy, and the anthropometric data of the newborn were data collected at the time of the questionnaire. At the time of birth, the information about the newborn was entered in the medical observation chart by the doctor (or midwife) who assisted the birth; these were also included in the questionnaire. For the newborn, low birth weight was <2500 g and low birth length was between 46 and 54 cm. This information was taken with the consent of the mother patient.

### 2.3. Statistical Analysis

The data were analyzed in association with the mother’s use of vitamin and folic acid supplements before and during her pregnancy. Additionally, the influence of certain socio-economic and behavioral factors on vitamin and folic acid supplementation was also assessed, before and during pregnancy, and weight gain during pregnancy according to the mother’s BMI (Body Mass Index) before pregnancy [20]. 

Depending on the result of checking the normality of the data, we applied parametric tests (unpaired t test) or non-parametric tests (Mann–Whitney test). To compare numerical data, we applied the Kruskal–Wallis test. The associations between socio-demographic factors, prenatal care, and vitamin intake before pregnancy and during pregnancy were assessed using the Chi-square test (χ^2^). We used logistic regressions to identify associations of vitamin intake before pregnancy and during pregnancy depending on infertility treatment, premature births, and low birth weight. 

A statistical analysis was performed using the Statistical Package for Social Sciences (SPSS software, version 22.0, Chicago, IL, USA), and the statistical significance threshold was set to 0.05. 

## 3. Results

From the 1278 women who initially filled in the questionnaire, 18.1% were excluded based on their reported irregular administration of vitamin supplements (there was no additional question to differentiate between irregular intake before pregnancy and during pregnancy; thus, we excluded these participants from the analysis). Out of the remaining 1047 interviewed post-partum women, 70.5% (738) reported not taking vitamin and folic acid supplements before pregnancy, while 31.2% (327) reported not taking vitamin supplements during pregnancy (Table S1). 

In Table 1, we tried to compare women who consumed vitamins both before pregnancy and during pregnancy with women who underwent infertility treatment, and who have a special status.

As shown in Table 2, the factors potentially influencing the consumption of vitamin supplements during pre-pregnancy and pregnancy were: the mother’s young age, the lack of prenatal visits to the physician, underweight before pregnancy, low weight gain during pregnancy, overweight before pregnancy, and increased weight gain during pregnancy.

As shown in Table 3, the average number of weeks of gestation was slightly increased in mothers with no pre-pregnancy vitamin supplementation compared to those who did not take vitamin supplements during pregnancy, with a similar situation observed in terms of the average weight of the newborns at birth.

In Table 4, we have identified, for women who want to become pregnant, what the risk factors are for vitamin consumption before pregnancy. Identified risk factors are: marital status (*p* < 0.001), level of education (*p* < 0.001), the fact that she has a job (*p* < 0.001), and even smoking (*p* = 0.001).

In Table 5 we identified, in pregnant women, what the risk factors are for vitamin consumption during pregnancy. Identified risk factors are: place of living (*p* = 0.02), marital status (*p* < 0.001), level of education (*p* < 0.001), the fact that she has a job (*p* < 0.001), and even smoking (*p* = 0.01).

It can be noticed that most of the risk factors are identical in both categories of women included in the study. During pregnancy, vitamin intake was identified as being significantly associated with their residential environment (*p* = 0.02) (Table 5).

The higher percentage of premature births and suboptimal anthropometric indices in the newborn were higher among women who did not take supplements before pregnancy compared to those who did not take supplements during pregnancy (Table 6).

Pregnancy weight gain above the optimal value of 16 kg was more frequent in pregnant women with a normal pre-pregnancy BMI, who consumed vitamin supplements both before and during pregnancy (Table 7). 

The absence of vitamin intake in the pre-pregnancy period was associated with the risk of premature birth (*p* = 0.01) and low birth weight (*p* = 0.01), and the absence of vitamin intake during pregnancy was associated with low birth weight of the newborn (*p* > 0.001) (Table 8).

A multivariate analysis identified a higher risk of vitamin deficiency in pregnant women who did not take vitamins before pregnancy, and premature birth and low birth weight were risks for women who did not take vitamin supplements during pregnancy (Table 9).

## 4. Discussion

Pre-conception care involves any intervention on the woman of reproductive age before pregnancy, with the aim of improving the health of the future mother, the newborn, and later even the child, with long-term implications. In addition, optimal nutritional support, with the onset of pregnancy, is very important for both the pregnant woman’s body and that of the fetus, which is developing.

Vitamin supplementation is frequently recommended during pregnancy [21,22,23]. A study conducted in Poland showed that the use of folic acid supplements was more common during pregnancy than in the pre-pregnancy period, and it was dependent on income, parity, and pregnancy planning [24].

In our study, fertility treatments, and supplementation before and after pregnancy were influenced by the mother’s age, marital status, and employment status (presence/absence of a job). The profile of the woman at risk of insufficient intake of nutrients needed during pregnancy is that of the mother who is too young, the woman who does not show up for prenatal visits, and the one with low body weight before pregnancy.

Women who reported taking vitamin supplements during pregnancy had a slightly longer gestation period and newborns with higher birth weights compared to women who did not take supplements during pregnancy.

Additionally, through our study we highlighted that the factors that influence the intake of vitamins before pregnancy are marital status, level of education, employment status, and the habit of smoking, while for pregnancy, the residential environment (urban/rural), is added to the other factors listed in the influence of vitamin intake.

Another prominent effect of vitamin intake is on the body weight of pregnant women. It has been observed that in women who had a normal BMI before pregnancy and consumed vitamins both before and during pregnancy, the weight gain during pregnancy was higher (>16 kg). For women who were overweight or obese before pregnancy, weight gain during pregnancy was above the normal limit, as opposed to taking vitamin supplements before or during pregnancy.

The absence of vitamin intake during pregnancy was associated with the risk of premature birth (*p* = 0.01) and low birth weight (*p* = 0.01) (Table 8), and the absence of vitamin intake during pregnancy was associated with low birth weight of the newborn (*p* < 0.001) (Table 8).

According to the data obtained by our study, we can say that women who do not take vitamin supplements are at a higher risk of premature birth or giving birth to a child with a lower birth weight. Regarding the height (length) of the newborn, apart from the optimal values, between 46 and 54 cm, we did not identify significant associations with vitamin supplementation.

In addition, women without vitamin supplements before pregnancy are more likely not to opt for vitamin supplements even during pregnancy, and the lack of vitamin supplements in pregnancy has exposed women to a higher risk of premature birth and low birth weight of the newborn.

In Norway, there is no mandatory fortification with folic acid, and women who intend to become pregnant have been advised since 1998 to take a daily supplement of 0.4 mg folic acid 1 month before conception and until 12 weeks of gestation to reduce the risk of neural tube defects (NTD) [1].

A study conducted in Romania between 2018 and 2019 shows that conception was spontaneous in 306 (89.5%) of the patients, and the rest had received in vitro fertilization (24 cases, 7%), or ovulation induction (11 cases, 3.2%). In total, 278 (83.5%) patients received folic acid throughout the first trimester of gestation [25].

Although the recommendation for supplementing vitamins and folic acid before and during pregnancy is generally accepted and applied, studies have shown that these recommendations are not followed by the target population worldwide [26,27]. In a group of women hospitalized in the post-partum period and studied in Berlin, Birkenberger et al. [28] found that two thirds of women did not use folic acid supplements during their pregnancy, especially those with a lower income level, lower level of education, at a very young age, or those who had an unplanned pregnancy. In addition, the rate of supplementation was lower among women from ethnic minorities. In our study, we included for pregnant women (including folic acid), and separately mentioned vitamin and folic acid consumption for women before pregnancy. A significant number of women (69.6%) did not take folic acid supplements before pregnancy. However, in comparison to the Birkenberger et al. study, only about a third (30.7%) of women refrained from taking vitamins during pregnancy. A possible explanation for the better compliance is that the percentage represents women from all groups, including those having a better education. 

Regarding the improvement of the consumption, Birkenberger et al. suggested teaching young subjects as part of their secondary education, with the help of Internet campaigns, in often visited public places, or by informing during visits at the gynecologist. Afterwards, there should be an evaluation of the efficiency of the information campaign. In this study, as well as in our own, one could not verify that the consumption of folic acid and vitamins was truly as declared by the participants. A validation of the declarations would be possible only through regular laboratory determinations of the values of interest. However, this can only be performed in a prospective study [28].

Another study from Norway showed a prevalence of only 16% of regular consumption of vitamin supplements in pregnancy, in a group of women interviewed immediately post-partum [29]. Estimates of prevalence for folic acid supplementation during the preconception period, collected by Toivonen and al. were 32–51% in North America, 9–78% in Europe, 21–46% in Asia, 4–34% in the Middle East, 32–39% in Australia/New Zealand, and 0% in Africa [30].

A study conducted in London showed that most women start using vitamin supplements only after the pregnancy has been confirmed, and only one third of them are supplementing their vitamin consumption before pregnancy [31]. In Romania, a study conducted in Iasi in 2010 on a group of pregnant women showed a prevalence of 48% for the use of folic acid supplements (during pregnancy), with an important percent (68%) of these women having a higher level of education [19].

Similar to the evidence found in the literature, our study also showed a statistically significant positive association between the lack of vitamin supplementation prior or during pregnancy, and a low birth weight, as well as a suboptimal gestational age at birth for the newborns [32]. Among the characteristics of the “risk profile” for lack of vitamin and folic acid supplements consumption, both before and during pregnancy, our study identified the following: very young mothers (adolescent), women who did not follow the scheduled visits to the family physician during the entire gestational period, low levels of weight gain during pregnancy, and rural residency. The pregnancy “risk profile” for insufficient vitamin consumption intake is similar to that described in other studies that addressed behavioral risk factors during pregnancy [33,34].

The limitations of this study would be the element of additional questions about vitamin intake during pregnancy. It would have been helpful to know how much folic acid they consumed before pregnancy or during pregnancy. There were no questions regarding the duration of consumption before pregnancy or the time during which pregnant women consumed folic acid (before birth or after birth). Another important limitation is that no information was requested about what vitamins were consumed during pregnancy (Fe, Iodine, D, etc.). Another limitation would be the women who reported an irregular consumption of folic acid, and whether this irregular consumption refers to the period before pregnancy or during pregnancy.

## 5. Conclusions

The consumption of vitamin supplements, including folic acid, is overall low in our studied female population before getting pregnant as well as during their pregnancy. This influences the optimal anthropometric parameters of the newborn, the normal gestational age at birth, and the weight gain during pregnancy according to the mother’s BMI before pregnancy. These aspects are further amplified by the presence of unfavorable socio-economic factors, low levels of education, and other risk factors related to lifestyle, such as smoking.

The fact that we observed the prevalence of this phenomenon of a lack of vitamin supplementation, even in women who are influenced by the analyzed risk factors, highlights the importance of promoting the roles of vitamin supplementation equally among all subjects. This would make it possible to avoid the targeted support of women belonging to a specific ethnic population, therefore eliminating a discriminatory approach. It is important to understand that a woman’s nutritional health must be ensured from the time before conception, both to create healthy habits to be followed during pregnancy and also for a successful pregnancy (birth outcome).

## Figures and Tables

**Table 1:** Basic characteristics of women interviewed about vitamin supplement use before and during pregnancy and fertility treatment, Târgu Mures, Romania, 2015–2016.

	Fertility Treatment	Supplement Use before Pregnancy	Supplement Use during Pregnancy	*p*
	(*n* = 66)	(*n* = 309)	(*n* = 720)	
Mother’s age (Mean/SD)	29.6/5.1	30.7/4.3	29.6/5.1	0.002 *
Mother’s BMI before pregnancy (Mean/SD)	23.8/5.2	22.1/3.3	22.3/3.6	0.19 *
Marital status Unmarried Married				<0.001 **
	6 (9.1%)	9 (2.9%)	93 (12.9%)	
	60 (90.9%)	300 (97.1%)	627 (87.1%)	
Workplace Unemployed Employed				<0.001 **
	15 (22.7%)	21 (6.8%)	132 (18.3%)	
	51 (77.3%)	288 (93.2%)	588 (81.7%)	
Coffee consumption Yes No				0.73 **
	36 (54.5%)	153 (49.5%)	357 (49.6%)	
	30 (45.5%)	156 (50.5%)	363 (50.4%)	
Alcohol consumption Yes No				0.054 **
	6 (9.1%)	15 (4.9%)	24 (3.3%)	
	60 (90.9%)	285 (92.2%)	696 (96.7%)	
Smoking Yes No				0.04 **
	9 (13.6%)	63 (20.4%)	180 (25.0%)	
	57 (86.4%)	246 (79.6%)	540 (75.0%)	

Study location Tirgu Mures, Romania, data collection 2015–2016. * Kruskal–Wallis test,** Chi-square test.

**Table 2:** Basic characteristics of women interviewed about vitamin supplement use before and during pregnancy, Tirgu Mures, Romania, 2015–2016.

Variables	Total (*n* = 1047)	No Supplement Use before or during Pregnancy (*n* = 738)	Supplement Use before and during Pregnancy (*n* = 309)	*p*	No Supplement Use in Pregnancy Only (*n* = 327)	Supplement Use in Pregnancy Only (*n* = 720)	*p*
	Mean SD	Mean SD	Mean SD		Mean SD	Mean SD	
Mother’s age	29.3	28.7	30.7	<0.001 *	28.6	29.6	0.02 **
	5.5	5.8	4.3		6.1	5.1	
Frequency of prenatal medical visits	8.7	8.5	9.2	0.16 **	7.8	9	>0.001 **
	4.4	4.5	4.2		4.78	4.2	
Mother’s BMI before pregnancy	22.3	22.4	22.1	0.10 **	27.7	22.3	0.63 **
	4.3	4.5	3.3		5	3.6	

Study location Tirgu Mures, Romania, data collection 2015–2016. * Unpaired *t* test; ** Mann–Whitney test.

**Table 3:** Vitamin supplementation associated with gestational age at birth and the newborn’s anthropometric measurements.

Variables	Total (*n* = 1047)	No Supplement Use before or during Pregnancy (*n* = 738)	Supplement Use before and during Pregnancy (*n* = 309)	*p*	No Supplement Use in Pregnancy Only (*n* = 327)	Supplement Use in Pregnancy Only (*n* = 720)	*p*
	Mean SD	Mean SD	Mean SD		Mean SD	Mean SD	
Gestational age at birth	38.2	38.2	38.2	0.06 *	37.9	38.3	0.01 *
	2.6	2.8	2.2		2.9	2.5	
Weight of newborn at birth (g)	3104.3	3075.9	3175.6	0.11 *	2999.4	3153.2	>0.001 *
	691.5	734	585		720.6	677.6	
Length of newborn at birth (cm)	51.8	51.9	51.8	0.21 *	51.8	51.8	0.92 *
	5.1	4.9	5.5		5.9	4.7	
Newborn’s head circumference at birth (cm)	33.9	33.7	34.4	0.31 *	33.4	34.2	0.10 *
	3.5	2.9	4.3		2.3	3.9	

Study location Tirgu Mures, Romania, data collection 2015–2016. * Mann–Whitney test.

**Table 4:** Vitamin supplementation pre-pregnancy as related to socio-economic status and behavioral factors with potential risks.

Variables	Absence of Vitamin and Folic Acid Supplementation before Pregnancy (*n* = 738)	Vitamin and Folic Acid Supplementation before Pregnancy (*n* = 309)	*p* OR 95%CI
Rural residence	311 (42.1%)	116 (37.5%)	0.167
Urban residence	427 (57.9%)	193 (62.5%)	1.21
			0.92–1.59
Married	564 (76.4%)	300 (97.1%)	<0.001
Unmarried	174 (23.6%)	9 (2.9%)	0.09
			0.04–0.19
High school	519 (70.3%)	290 (93.9%)	<0.001
Less than high school	119 (16.1%)	19 (6.1%)	0.28
			0.17–0.47
Employed	525 (71.1%)	21 (6.8%)	<0.001
Unemployed	213 (28.9%)	288 (93.2%)	33.8
			21.10–54.13
Coffee consumption			0.35
Yes	384 (52.0%)	153 (49.5%)	1.13
No	345 (46.7%)	156 (50.5%)	0.86–1.48
Alcohol consumption			0.34
Yes	27 (3.7%)	15 (4.9%)	0.73
No	699 (94.7%)	285 (92.2%)	0.38–1.40
Smoking			0.001
Yes	222 (30.1%)	63 (20.4%)	1.68
No	516 (69.9%)	246 (79.6%)	1.22–2.31

Study location Tirgu Mures, Romania, data collection 2015–2016.

**Table 5:** Vitamin supplementation during pregnancy as related to socio-economic status and behavioral factors with potential risks.

Variables	Absence of Vitamin Supplementation during Pregnancy (*n* = 327)	Vitamin Supplementation during Pregnancy (*n* = 720)	*p* OR 95%CI
Rural residence	150 (45.9%)	277 (38.5%)	0.02
Urban residence	177 (54.1%)	443 (61.5%)	1.35
			1.04–1.76
Married	237 (72.5%)	627 (87.1%)	<0.001
Unmarried	90 (27.5%)	93 (12.9%)	0.39
			0.28–0.54
High school	199 (60.9%)	610 (84.7%)	<0.001
Less than high school	128 (39.1%)	110 (15.3%)	0.28
			0.20–0.37
Employed	207 (63.3%)	588 (81.7%)	<0.001
Unemployed	117 (35.8%)	132 (18.3%)	0.39
			0.29–0.53
Coffee consumption			0.07
Yes	180 (55.0%)	357 (49.6%)	1.27
No	144 (44.0%)	363 (50.4%)	0.97–1.65
Alcohol consumption			0.09
Yes	18 (5.5%)	24 (3.3%)	1.7
No	306 (93.6%)	696 (96.7%)	0.91–3.19
Smoking			0.01
Yes	105 (32.1%)	180 (25.0%)	1.41
No	222 (67.9%)	540 (75.0%)	1.06–1.89

**Table 6:** Relationship between vitamin supplementation before and during pregnancy and parameters related to getting pregnant, pregnancy evolution, and birth outcomes.

Variable	Absence of Vitamin and Folic Acid Supplementation before Pregnancy (*n* = 738)	Absence of Vitamin Supplementation during Pregnancy (*n* = 327)
Under 36 weeks at birth	87 (11.7%)	38 (11.6%)
Low birth weight	114 (15.4%)	63 (19.3%)
Low birth length	60 (8.1%)	24 (7.3%)

Study location Tirgu Mures, Romania, data collection 2015–2016.

**Table 7:** Weight gain of the mother based on vitamin supplements intake, before and during pregnancy.

Mother’s BMIbefore Pregnancy	Weight Gain during Pregnancy According to the Mother’s BMI before Pregnancy	Absence of Vitamin and Folic Acid Supplementation before Pregnancy (*n* = 738)	Vitamin and Folic Acid Supplementation before Pregnancy (*n* = 309)	Absence of Vitamin Supplementation during Pregnancy (*n* = 327)	Vitamin Supplementation during Pregnancy (*n* = 720)
<18.5 (underweight)	<12.5 kg	39	6	33 (10.1%)	18
	12.5–18 kg	47 (6.3%)	9	18	38 (5.3%)
	>18 kg	15	12 (3.9%)	9	18
18.5–24.9 (normoponderal)	<11.5 kg	163 (22.0%)	42	79 (24.2%)	126
	11.5–16 kg	161	87	60	188
	>16 kg	152	102 (32.7%)	45	205 (28.47%)
25.0–29.9 (overweight)	<7 kg	11	0	10	1
	7–11.5 kg	28	15	7	36
	>11.5 kg	76 (10.3%)	33 (10.6%)	51 (15.6%)	57 (7.9%)
≥30.0 (obese)	<5 kg	3	0	3	0
	5–9 kg	7	0	1	6
	>9 kg	33 (4.4%)	6 (1.9%)	11 (3.4%)	27 (3.8%)

Study location Tirgu Mures, Romania, data collection 2015–2016.

**Table 8:** Association of supplements intake before and during pregnancy.

	Absence of Vitamin and Folic Acid Supplementation before Pregnancy (*n* = 738)	Vitamin and Folic Acid Supplementation before Pregnancy (*n* = 309)	*p* RR 95%CI	Absence of Vitamin Supplementation during Pregnancy (*n* = 327)	Vitamin Supplementation during Pregnancy (*n* = 720)	*p* RR 95%CI
Under 36 weeks at birth	87 (11.8%)	21 (6.8%)	0.01	38 (11.6%)	70 (9.7%)	0.38
Over 36 weeks at birth	651 (88.2%)	288 (93.2%)	1.73	289 (88.4%)	650 (90.3%)	1.19
			1.09–2.74			0.82–1.73
Low birth weight	114 (15.4%)	30 (9.7%)	0.01	63 (19.3%)	81 (11.3%)	<0.001
More birth weight	621 (84.1%)	276 (89.3%)	1.58	261 (79.8%)	636 (88.3%)	1.72
			1.08–2.31			1.27–2.32
Low birth length	60 (8.1%)	30 (9.7%)	0.46	24 (7.3%)	69 (9.6%)	0.34
More birth length	660 (89.4%)	276 (89.3%)	0.85	288 (88.1%)	645 (89.6%)	0.79
			0.56–1.29			0.51–1.24

Study location Tirgu Mures, Romania, data collection 2015–2016.

**Table 9:** Logistic regression model for the association of supplements intake in pre-pregnancy and pregnancy period.

	Absence of Vitamins Supplementation and Folic Acid before Pregnancy (*n* = 738) versus Vitamins Supplementation and Folic Acid before Pregnancy (*n* = 309)			Absence of Vitamins Supplementation during Pregnancy (*n* = 327) versus Vitamins Supplementation during Pregnancy (*n* = 720)		
	OR	95% CI	*p*	OR	95% CI	*p*
No vitamins and folic acid intake before pregnancy				2.25	1.60–3.16	<0.0001 *
Under 36 weeks at birth	1.77	0.88–3.53	0.10	0.45	0.24–0.83	0.01 *
Low birth weight	1.11	0.66–1.87	0.67	2.42	1.55–3.79	0.001 *
Low birth length	0.90	0.66–1.21	0.50	1.08	0.80–1.45	0.59
No vitamin intake during pregnancy	2.25	1.60–3.16	<0.001 *			

Study location Tirgu Mures, Romania, data collection 2015–2016. * Statistical significance *p* < 0.05.

## Supplementary Materials

The following supporting information can be downloaded at: https://www.mdpi.com/article/10.3390/ijerph19148503/s1. Table S1: Basic characteristics about vitamin supplement use before and during pregnancy, Tirgu Mures, Romania, 2015–2016.
