# Supplementary material for: Vitamin Supplementation in Pre-Pregnancy and Pregnancy among Women—Effects and Influencing Factors in Romania
Source: Int J Environ Res Public Health. 2022 Jul 12;19(14):8503. doi: 10.3390/ijerph19148503 (PMC9318761; doi:10.3390/ijerph19148503)
Supplement: Supplementary file 1 [file ijerph-19-08503-s001.zip › ijerph-1694027-supplementary.pdf]

Table S1. Basic characteristics about vitamin supplement use before and during pregnancy, Tirgu Mures, Romania, 2015-2016

		Supplement use during pregnancy		
		No	Yes	
Supplement use before pregnancy	No	264	63	p<0.001*
	Yes	474	246	

*Chi-square test
